# Phase II study evaluating consolidation whole abdominal intensity-modulated radiotherapy (IMRT) in patients with advanced ovarian cancer stage FIGO III - The OVAR-IMRT-02 Study

**DOI:** 10.1186/1471-2407-11-41

**Published:** 2011-01-28

**Authors:** Nathalie Rochet, Meinhard Kieser, Florian Sterzing, Sonja Krause, Katja Lindel, Wolfgang Harms, Michael H Eichbaum, Andreas Schneeweiss, Christof Sohn, Juergen Debus

**Affiliations:** 1 Department of Radiation Oncology, University of Heidelberg, Im Neuenheimer Feld 400, 69120 Heidelberg, Germany; 2 Institute of Medical Biometry and Informatics, University of Heidelberg, Im Neuenheimer Feld 305, 69120 Heidelberg, Germany; 3 Department of Gynaecology and Obstetrics, University of Heidelberg, Voss Strasse 9, 69115 Heidelberg, Germany; 4 National Center for Tumor Diseases, University of Heidelberg, Im Neuenheimer Feld 460, 69120 Heidelberg, Germany

## Abstract

**Background:**

The prognosis for patients with advanced FIGO stage III epithelial ovarian cancer remains poor despite the aggressive standard treatment, consisting of maximal cytoreductive surgery and platinum-based chemotherapy. The median time to recurrence is less than 2 years, with a 5-years survival rate of -20-25%. Recurrences of the disease occur mostly intraperitoneally.

Ovarian cancer is a radiosensitive tumor, so that the use of whole abdominal radiotherapy (WAR) as a consolidation therapy would appear to be a logical strategy. WAR used to be the standard treatment after surgery before the chemotherapy era; however, it has been almost totally excluded from the treatment of ovarian cancer during the past decade because of its high toxicity. Modern intensity-modulated radiation therapy (IMRT) has the potential of sparing organs at risk like kidneys, liver, and bone marrow while still adequately covering the peritoneal cavity with a homogenous dose.

Our previous phase I study showed for the first time the clinical feasibility of intensity-modulated WAR and pointed out promising results concerning treatment tolerance. The current phase-II study succeeds to the phase-I study to further evaluate the toxicity of this new treatment.

**Methods/design:**

The OVAR-IMRT-02 study is a single-center one arm phase-II trial. Thirty seven patients with optimally debulked ovarian cancer stage FIGO III having a complete remission after chemotherapy will be treated with intensity-modulated WAR as a consolidation therapy.

A total dose of 30 Gy in 20 fractions of 1.5 Gy will be applied to the entire peritoneal cavity including the liver surface and the pelvic and para-aortic node regions. Organ at risk are kidneys, liver (except the 1 cm-outer border), heart, vertebral bodies and pelvic bones.

Primary endpoint is tolerability; secondary objectives are toxicity, quality of life, progression-free and overall survival.

**Discussion:**

Intensity-modulated WAR provides a new promising option in the consolidation treatment of ovarian carcinoma in patients with a complete pathologic remission after adjuvant chemotherapy. Further consequent studies will be needed to enable firm conclusions regarding the value of consolidation radiotherapy within the multimodal treatment of advanced ovarian cancer.

**Trial registration:**

Clinicaltrials.gov: NCT01180504

## Background

Ovarian cancer has the highest mortality rate of all gynaecologic cancer in the western world. Symptoms tend to be vague; hence the disease is often detected late. 75% of patients present with advanced stage disease, after it has spread into the peritoneal cavity (stage FIGO III). Despite the aggressiveness of the current standard treatment, consisting in maximal cytoreductive surgery and adjuvant combination chemotherapy (carboplatin and taxane), the prognosis for patients in a stage FIGO III disease remains poor. The median time to recurrence is less than 2 years. Typically recurrences occur intraperitoneally, distant metastasis appear mostly later in the course of the disease. The 5-year survival rate for stage FIGO III disease is approximately 20-25% [1-5]. Various sorts of consolidation therapy have been studied in a series of clinical trials, but yet none has shown to improve survival in ovarian cancer [6-8].

The use of whole abdominal radiotherapy (WAR) as a consolidation therapy would appear to be a logical strategy given that ovarian cancer is a radiosensitive tumor. The fact that WAR can be curative in certain groups of patients has been shown by intensive studies by Dembo et al [9,10]. However, the role of radiation therapy in ovarian cancer remains controversial. In the 1950s, surgery and adjuvant WAR were the dominant treatment modalities [9,10]. Since the improvements of chemotherapy, which gives primarily high response rates and has a systemic effect, radiotherapy has been increasingly abandoned [11].

WAR as a consolidation concept after surgery and chemotherapy has been evaluated in a number of small and non-randomized studies with contradictory results [12-17]. The recent randomized trial of Sorbe indicated that WAR may have a place in the treatment of advanced ovarian carcinoma in patients with complete pathologic remission after adjuvant chemotherapy [18]. A potential benefit was noted regarding the rate of recurrence and progression-free as well as overall survival rates. These results are supported by other small recent series [15,19-23]. It seems that 30 Gy delivered to the whole abdomen can eliminate residual microscopic disease in 45-50% of the cases [15].

However, there are many reasons why consolidation WAR fell into disfavour. It is associated with a high rate of severe acute side effects, particularly myelosuppression, due to additive toxicities after chemotherapy, which often leads to delayed or incomplete radiotherapy [16]. WAR is ineffective for patients with macroscopic disease residuum after chemotherapy [24]. Using the conventional technique, WAR is not able to deliver adequate radiation doses to the upper abdomen, due to limiting organs at risk (OAR), especially liver and kidneys [9]. Eventually WAR is unpopular because of the late bowel toxicity, which is surgically difficult to manage.

As a consequence, during the past decade, WAR has been almost totally excluded from the treatment of advanced ovarian. However, toxicity can now be managed by modern radiotherapy techniques using intensity-modulated radiotherapy (IMRT), which allow a very precise irradiation of complex target volumes while sparing organs at risk like kidneys, liver, and bone marrow [25-29].

Our previous phase I study showed, for the first time, the clinical feasibility of intensity-modulated WAR in combination with modern chemotherapy and surgery and pointed out promising results concerning treatment tolerance [26,30,31]. Ten patients with optimally debulked ovarian cancer FIGO stage IIIc were treated with intensity-modulated WAR up to a total dose of 30 Gy in 1.5 Gy fractions as a consolidation therapy after adjuvant carboplatin/taxane chemotherapy. Treatment was delivered using IMRT in step-and-shoot technique (n = 3) or helical tomotherapy technique (n = 7). The planning target volume (PTV) included the entire peritoneal cavity and the pelvic and para-aortic node regions. Organs at risk (OARs) were kidneys, liver, heart, vertebral bodies and pelvic bones.

Intensity-modulated WAR rendered an excellent coverage of the entire peritoneal cavity including frequent sites of abdominal recurrence like the liver capsule and the diaphragm as well as of the pelvic- and para-aortic node regions. An effective sparing of liver, kidneys and bone marrow allowed delivering the treatment as planned, with a high compliance of patients. Radiotherapy was well tolerated, no severe acute side effects CTC grade 4 occurred. CTC grade 3 toxicities were: diarrhea (n = 1), thrombocytopenia (n = 1) and leukopenia (n = 3). After a median follow-up of 23 months, 4 patients had a tumor recurrence (intraperitoneal progression n = 3, hepatic metastasis n = 1). Small bowel obstruction due to adhesions occurred in 3 patients, 2.5, 3 and 13 months respectively after radiotherapy.

We initiated the current phase-II study to further evaluate toxicity and outcome of intensity-modulated whole-abdominal radiotherapy. The primary objective of the OVAR-IMRT-02 trial is to demonstrate the good tolerability this intensive multimodal adjuvant treatment in a cohort of 37 patients with advanced ovarian-cancer FIGO stage III, optimally debulked and with a complete remission after chemotherapy. Secondary objectives are rate of therapy stop, rate of therapy interruption, rate of acute toxicity, rate of late toxicity, rate of relapse-free survival, rate of overall-survival and quality of life (EORTC-QLQ30).

## Methods/design

### Study design

The OVAR-IMRT-02 is designed as a single-center one arm prospective phase II trial. In order to accelerate the data collection we plan to associate a second study center. Patients fulfilling the inclusion criteria will be treated with intensity-modulated WAR up to a total dose of 30 Gy in 1.5 Gy fractions within a consolidation concept. Thirty seven patients are planned to be enrolled.

### Study objectives

The aim of the study is to demonstrate superior tolerability of consolidating intensity-modulated WAR compared to the conventional technique.

To get an impression of the tolerability of current radiation approaches, we explored the results from historical data on conventional radiation procedure. Adjuvant WAR using the conventional technique after radical surgery and chemotherapy showed a high rate of acute side effects (especially hematotoxicity, but also nausea, vomiting and diarrhoe). The pronounced acute toxicity, which is described in the literature as "severe", leads in many cases to a stop (10-15%) or to an interruption (15-30%) of the therapy and with it to a reduction of its efficacy [22,23,32,33]. This is one of the reasons why WAR is no longer applied. Within the OVAR-IMRT-01 Study, none of the 10 patients showed an acute toxicity CTC grade 4 (0%), and the CTC grade 3 toxicity rate was 40%.

Primary outcome variable of the OVAR-IMRT-02 Study is the tolerability of therapy, defined by the non-occurrence of CTC grade 4 toxicity during the radiation therapy and until 6 weeks after its termination.

Secondary outcome variables are the rate of therapy stop, rate of therapy interruption, rate of acute toxicity, rate of late toxicity, rate of relapse-free survival, rate of overall survival and quality of life (EORTC-QLQ30).

### Trial organisation

The trial OVAR-IMRT-02 is an investigator initiated trial. The trial is carried out by the Department of Radiation Oncology at the University of Heidelberg in co-operation with the Department of Gynaecology and Obstetrics. Statistical analysis and data management are performed by the Institute of Medical Biometry and Informatics at the University of Heidelberg. The trial is partly financially supported by the German Cancer Aid (Deutsche Krebshilfe).

### Coordination

The overall coordination is performed by the Department of Radiation Oncology at the University of Heidelberg.

### Patient selection: Inclusion criteria

Patients meeting all the following criteria will be considered for admission to the trial:

• histologically confirmed ovarian cancer or tube cancer or primary peritoneal carcinoma stage FIGO III

• status post primary optimal debulking surgery

• postoperative gross residual tumor ≤ 1 cm (R0, R1 or R2 < 1 cm situation)

• status post (neo-) adjuvant chemotherapy with platin and taxane

• complete remission after adjuvant chemotherapy (clinically, radiographically and biochemically)

• Karnofsky performance score > 60

• patient >18 years of age

• written informed consent

### Patient selection: Exclusion criteria

• stage FIGO I or II

• stage III with postoperative gross residual tumor > 1cm

• stage FIGO IV

• recurrence situation

• delayed wound healing post laparotomy

• leucopenia <2000/ml before radiotherapy

• thrombocytopenia <75000/ml before radiotherapy

• clinically active renal, hepatic, cardiac, metabolic, respiratory, coagulation or hematopoietic disease

• status post pelvic or abdominal radiotherapy

• status post other cancer disease in the past 5 years (cervical cancer in situ, basal cell carcinoma, squamous cell carcinoma of the skin are excluded)

• participation in another clinical trial

## Statistical Design and Methods

The primary endpoint of the trial is the tolerability rate, which is calculated as the percentage of patients for which no CTC Grade 4 toxicity occurs during the radiation therapy and until 6 weeks after its termination. The target tolerability rate is 90%, and a tolerability rate of less or equal to 70% is considered to be not of interest. Denoting the tolerance rate by *p*, the related test problem can be formulated as *H*_*0*_: *p *≤ *p*_*0 *_= 0.70 versus *H*_1_: *p *≥ *p*_1 _= 0.90 which will be assessed at one-side significance level *α *= 0.05.

The trial is designed as a two-stage study with a planned interim analysis, allowing for early stopping, and a power of 0.90 to reject the null hypothesis. In the first stage, 15 patients are enrolled. If all these patients would tolerate radiation therapy according to the definition of the primary endpoint, the study would stop with rejection of the null hypothesis. The study would terminate with acceptance of the null hypothesis, if less than 12 of the first 15 patients would tolerate the radiation therapy. According to the current design, the trial would continue with the second stage enrolling further 22 patients, if the number of patients that tolerate radiation therapy is between 12 and 14. In the final analysis, the null hypothesis can be rejected if the product of the one-sided p-values of the two stages is lower or equal to 0.021. With an expected sample size of 21.4 for a tolerability rate of *p*_0 _= 0.70 (i.e., under the null hypothesis), this design shows similar characteristics as Simon's (1989) optimal design whose expected sample size is 21.2. under the same scenario [34]. However, the adaptive two-stage design implemented in this trial allows a data-driven modification of the sample size of the second stage based on the results of the interim analysis or on external information while the specified significance level is controlled when the decision rules defined above are adhered to [35].

Additionally to the hypothesis testing approach, the primary endpoint will be analysed by applying Bayesian methods. Starting with the uniform distribution for the tolerability rate as prior distribution for the preceding trial OVAR-IMRT-01 [26,30,31], the posterior distribution of this trial is derived which serves as prior distribution for the current study OVAR-IMRT-02. Employing the results of OVAR-IMRT-02 leads to an updated posterior distribution for the tolerability rate from which Bayesian inference on *p *will be derived.

The secondary outcome variables will be evaluated by application of methods of descriptive data analysis. Appropriate measures of the empirical distribution as well as 95% confidence intervals will be calculated. All statistical analyses will be performed with SAS version 9.0 or higher.

### Investigation schedule

#### Therapy Indication

For each patient, the oncologic treatment concept after diagnosis is based on a tumor board review, following approved standard therapies and current guidelines. Consent of the colleagues from gynaecology, internal medicine and radiation oncology are obligatory.

#### Pre-therapeutic examinations

A complete staging is performed before surgery and/or chemotherapy, including abdominopelvic CT scan or MRI, radiography or CT scan of the chest, liver sonography, pelvic examination, transvaginal sonography, CA-125. After completion of chemotherapy, the complete remission is assessed by pelvic examination, transvaginal sonography and measure of CA-125.

The baseline visit is performed at study enrollment, after obtaining of the written consent of the patient. It includes blood test, clinical examination and quality of life questionnaire (EORTC-QLQ30).

#### Radiotherapy-planning

After immobilisation in a vacuum mattress and a scotch cast head mask (arms above the head) the patients undergo a native and contrast enhanced abdomino-pelvic computed tomography (optionally with 4D respiratory triggering). This CT scan is used for the restaging and for the radiation planning.

The clinical target volume (CTV) includes the whole peritoneal cavity extending from diaphragm to douglas cavity, the liver surface, and the pelvic and para-aortic node regions. The planning target volume (PTV) encompasses an axial margin of 1.5 cm around the CTV (2.5 cm in the cranial direction). Organs at risk are kidneys, liver (except the 1 cm-outer border), lungs, bones (vertebral bodies and pelvic bones), hear and spinal cord.

A dose of 30.0 Gy is prescribed to the median of the PTV. Inverse radiation planning is performed according to the national DIN norm and international recommendations (ICRU 50 Report 1999). The goal of optimization is to deliver a dose distribution in the PTV as homogeneous as possible in spite of maximal sparing of OARs with a high priority on liver, kidney and bone marrow. Tolerated maximum doses to OARs are not to exceed the TD5/5 for each organ (31).

#### Radiation therapy

Irradiation is applied as helical intensity-modulated radiation therapy (IMRT) using a tomotherapy device (Madison, Wisconsin, USA) with 6 MeV photons. Control of positioning accuracy is performed daily with the integrated megavoltage computed tomography for tomotherapy (MV-CT scan, 3.5 MV).

#### Duration of treatment

The delay between the last chemotherapy and the onset of radiation should not exceed 10 weeks. The duration of the treatment planning is estimated about 2 or 3 weeks. The duration of radiotherapy is 4 weeks (20 fractions). The overall daily treatment time is expected to be about 15 to 20 minutes with a mean daily "time on table" of about half an hour.

#### Treatment schedule

The radiotherapy is performed on an out-patient basis with the possibility of hospital admission if required.

#### Monitoring during treatment

Patients are evaluated weekly during radiotherapy. Antiemetic and antidiarrheal medications are prescribed as required. Blood count is assessed twice weekly. Toxicities are graded according to the National Cancer Institute Common Toxicity Criteria version 3.0. The quality of life is assessed at the last treatment fraction using EORTC-QLQ30 questionnaire. The documentation of acute hematologic, genito-urinary and gastro-intestinal acute toxicities is especially relevant.

### Follow up

Follow-up visits are scheduled in addition to the standard gynaecology follow-up program at 6 weeks, 3, 6, 9, 12, 15, 18, 24, 30 and 36 months, respectively, after completion of radiotherapy. Each visit includes blood test, CA-125, pelvic examination, transvaginal ultrasound, recording of adverse events and assessment of quality of life (questionnaire EORTC-QLQ30). The documentation of late genito-urinary and gastro-intestinal toxicities (such as bowel obstruction) is especially relevant. Additionally, a contrast-enhanced abdomino-pelvic CT scan or MRI will be performed at 6, 12 und 24 months, respectively.

### Duration of the study

The primary objective of the study is to prove the good treatment tolerance, defined as non-occurrence of CTC grade 4 toxicities during radiotherapy and till 6 weeks after its completion. To assess the primary endpoint, the final study visit will be 6 weeks after the last patient completed the radiotherapy. The study ends three years after the last patient was treated. Recruitment of the patients is planned over a time period of 36 months; follow-up duration will be 36 months.

### Data Handling, Storage and Archiving of Date

All findings including clinical and laboratory data will be documented by the investigator or an authorized member of the study team in the subject's medical record and in the CRF. The data will be stored and archived according to the §13 of the German GCP-Regulation and §28 c of the German X-Ray Regulation (StrlSchV) for at least 30 years after the trial termination.

### Ethics, informed consent and safety

The final protocol was approved by the ethics committee of the University of Heidelberg, Heidelberg, Germany (Nr: S-353/2008). Additionally, a positive vote of the Bundesamt für Strahlenschutz (BfS) has been obtained. This study complies with the Helsinki Declaration in its recent German version. The trial will also be carried out in keeping with local legal and regulatory requirements. The ClinicalTrials.gov Protocol ID is NCT01180504.

All severe adverse events have to be reported immediately to the principal investigator. In addition, side effects and patient safety will be monitored by an independent data safety monitoring committee (DMC), composed of one radio oncologist, one gynaecologist and one statistician. In case of frequent or particularly serious adverse events, then the DMC would have to consider termination of the study. This evaluation has to be made in consideration of risk/benefit. The DMC will meet on a regular basis.

## Discussion

The prognosis for patients with advanced FIGO stage III epithelial ovarian cancer remains poor despite the aggressive standard treatment, consisting of maximal cytoreductive chemotherapy and platinum-based chemotherapy. The median time to recurrence, which occurs mostly intraperitoneally, is less than 2 years, with a 5-years survival rate of -20-25% [1-5].

Ovarian cancer is a radiosensitive tumor, so that WAR as a consolidation therapy would appear to be a logical strategy. WAR used to be the standard treatment after surgery before the chemotherapy era. However, WAR has been almost totally excluded from the treatment of ovarian cancer during the past decade because of its high toxicity [9,10]. Since toxicity can be managed by modern radiotherapy technique (IMRT), WAR provides a new promising option in the consolidation treatment of ovarian carcinoma in patients with complete pathologic remission after adjuvant chemotherapy [26,30].

In our previous phase-I study, we showed for the first time the clinical feasibility of intensity-modulated WAR in combination with modern chemotherapy and surgery [31]. It rendered an excellent coverage of the entire peritoneal cavity including frequent sites of abdominal recurrence like the liver capsule and the diaphragm as well as of the pelvic- and para-aortic node regions. An effective sparing of liver, kidneys and bone marrow allowed delivering the treatment as planned, with a high compliance of patients.

The current phase-II succeeds to the phase-I study to demonstrate the good tolerability of the treatment (defined as non-occurrence of CTC grade 4 acute toxicities) and to further evaluate toxicity and outcome of this new treatment.

In the previous phase-I study, small bowel obstruction due to adhesions occurred in 3 of 10 patients, 2.5, 3 and 13 months respectively after radiotherapy. The exact role played by radiotherapy in the development of peritoneal adhesions remains unclear and further studies are needed to evaluate this problem. Risk factors for late bowel toxicity are suggested by literature: dose escalation with a total abdominal dose over 30 Gy, pelvic-boost and/or para-aortic boost and previous second-look laparotomy are associated with a significant increase in small bowel obstruction [23,32,33]. Given that the addition of pelvic boost treatment is not clearly beneficial in the literature; the routine use of a pelvic boost in consolidation WAR should be questioned [23]. With respect to these arguments, this study was designed with careful attention to keeping the risk for late bowel toxicity as low as possible and bowel adverse events will be monitored accurately.

Intensity-modulated WAR could offer a new therapeutic option for consolidation treatment of advanced ovarian carcinoma after adjuvant chemotherapy in selected subgroups of patients. However, further consequent studies are needed to draw firm conclusions regarding the value of consolidation radiotherapy in the multimodal treatment of ovarian carcinoma, including immunologic and biologic therapies in the future.

## Abbreviations

CRF: Case Report Form; CTCAE: Common Terminology Criteria for Adverse Events; CTV: Clinical Target Volume; DMC: Data Monitoring Committee; FIGO: International Federation of Gynaecology and Obstetrics; GCP: Good Clinical Practice; IMRT: Intensity Modulated Radiation Therapy; PTV: Planning Target Volume; TD5/5: Tolerance Dose 5% over 5 years; WAR: Whole-Abdominal Radiotherapy

## Competing interests

The authors declare that they have no competing interests.

## Authors' contributions

NR, MK, AS, WH and JD planned, organized and conduct the study. NR, KL, ME, AS, and CS recruit the patients for the study. NR, FS and SK perform planning and radiation therapy. Medical care and follow up is provided by NR, FS, SK, KL, ME, AS and CS. Statistical analysis is carried out by MK. All authors have read and approved the final manuscript.

## Pre-publication history

The pre-publication history for this paper can be accessed here:

http://www.biomedcentral.com/1471-2407/11/41/prepub
